# Preparation of Honey-Loaded Liposomes and Evaluation of Their Physicochemical and Antibacterial Properties

**DOI:** 10.5812/ijpr-158198

**Published:** 2026-07-18

**Authors:** Zahra Nazemoroaya, Arash Mahboubi, Hamid Reza Moghimi

**Affiliations:** 1 Department of Pharmaceutics and Pharmaceutical Nanotechnology, School of Pharmacy, Shahid Beheshti University of Medical Sciences, Tehran, Iran; 2 Food Safety Research Center, Shahid Beheshti University of Medical Sciences, Tehran, Iran; 3 Protein Technology Research Center, Shahid Beheshti University of Medical Sciences, Tehran, Iran

**Keywords:** Honey, Liposomes, Characterization, Antibacterial Activity, Release Kinetics

## Abstract

**Background:**

Honey is a natural wound-healing agent with antibacterial properties. It is applied to wounds in pure form or in conventional formulations; however, its application and efficacy may be challenging. Liposomes have been used as promising carriers for natural products. Therefore, honey-loaded liposomes were prepared and investigated as novel carriers for honey.

**Objectives:**

This study aimed to prepare honey-loaded liposomes using different honey concentrations and to characterize the formulations.

**Methods:**

Honey-loaded liposomes were prepared using the thin-film hydration method with egg lecithin and cholesterol at an 8:2 molar ratio and were hydrated with aqueous solutions containing 5% – 75% (w/v) honey. The mean particle size and zeta potential were measured by dynamic light scattering. Surface morphology was characterized by electron microscopy. Entrapment efficiency and the percentage of honey released were evaluated by quantifying honey polyphenols using the Folin-Ciocalteu assay as a marker of honey content. The minimum inhibitory concentration and minimum bactericidal concentration against *Staphylococcus aureus* and *Pseudomonas aeruginosa* were determined using a microdilution method.

**Results:**

The liposomes had particle sizes of 94 - 282 nm, with the highest initial honey concentration (75%) yielding the largest particle size. Entrapment efficiencies were 22%–46%, with the 50% initial honey concentration showing the highest value. Increasing the initial honey concentration increased the honey content in the formulations. The zeta potential of plain liposomes was approximately -16 mV and shifted toward neutral values in honey-loaded liposomes (approximately -8 to -4 mV). Honey-loaded liposomes appeared as spherical particles, with some distorted spherical/angular particles. Honey release from liposomes showed a more extended pattern than that of honey solutions and followed Fickian diffusion kinetics from spheres for liposomes prepared with 5%–50% honey concentrations, whereas liposomes with a 75% initial honey concentration apparently favored first-order release kinetics. Biological assays showed enhanced antibacterial effects of liposomes of up to 3.5-fold and 7-fold against *Staphylococcus aureus* and *Pseudomonas aeruginosa*, respectively, compared with honey. The liposomal formulation with 75% initial honey showed the highest antibacterial activity.

**Conclusions:**

The present results demonstrate that liposomes can deliver honey compounds and that honey-loaded liposomes may be promising candidates for medical applications, such as wound treatment.

## 1. Background

The emergence of antibiotic resistance, coupled with the declining efficacy of existing antibiotics, has prompted scientists to explore alternative therapeutic options derived from natural sources (1). These alternatives offer several benefits, including synergistic effects, efficacy, availability, low cost, and excellent biocompatibility (2). Honey, a natural substance, has a long history of use in medicinal practice. The therapeutic benefits of honey are attributed to several factors, including its high osmolarity, acidic nature, generation of hydrogen peroxide during glucose oxidation, and abundance of beneficial bioactive components, including phenolic compounds (3).

Owing to its physical properties, honey is difficult to apply directly to wound sites (4). In addition, the limited bioavailability of active constituents, such as phenolic compounds, restricts their effectiveness under in vitro and in vivo conditions (5). In general, honey is not used as an antibacterial agent for systemic infection therapy; it must be in contact with the target microorganism and is therefore used as a topical treatment for wound infections (6).

The complex composition of natural products presents a major challenge in the development of drug-delivery systems. Although different scaffolds containing honey, such as hydrogels and nanofibers, are widely used (3, 7), few studies have examined honey-loaded nanoparticles. Recent studies indicate that combining meropenem and honey within chitosan nanoparticles can enhance antibacterial and antibiofilm properties and reduce antibiotic cytotoxicity (8). Furthermore, encapsulating honey sourced from the Himalayan mountains into iron oxide nanoparticles significantly enhances its antimicrobial and antioxidant activities compared with free Himalayan honey (9). These studies highlight the unique properties of honey in combination with nanotechnology and suggest promising approaches for improving its antioxidant and antimicrobial effects.

In recent years, different types of lipid nanocarriers, including liposomes, have been investigated and used to improve the efficacy of therapeutic agents, with extensive applications in the pharmaceutical and cosmetic industries (10, 11). These lipid-based carriers have attracted attention because of their ability to encapsulate various substances in both aqueous and lipid phases. Liposomes offer additional advantages, including enhanced drug penetration, improved bioavailability, controlled drug release, and reduced adverse effects (12, 13). Consequently, liposomes serve as versatile vehicles for encapsulating a wide range of natural substances (13).

## 2. Objectives

Despite extensive research on liposomes, to the best of our knowledge, no study has investigated honey-loaded liposomes; however, some studies have examined propolis (14) and honey components, such as methylglyoxal found in Manuka honey (15). Given the advantageous structural properties of liposomal formulations and the promising therapeutic attributes of honey for infection treatment, this study aimed to develop honey-loaded liposomes and evaluate their antibacterial activity. To this end, different concentrations of honey were incorporated into the liposomal system, and the effects of these honey-loaded liposomes on parameters such as entrapment efficiency, release profiles, release kinetics, and surface morphology were investigated. In addition, the antibacterial performance of these liposomes against *Pseudomonas aeruginosa* and *Staphylococcus aureus* was evaluated. This research provides valuable insights into the use of liposomal carriers for honey-based therapies and highlights their potential for drug delivery and infection management systems.

## 3. Methods

### 3.1. Materials

Honey was sourced from a local beekeeper in northern Iran. Egg phosphatidylcholine (egg PC) (Lipoid E80; PC > 80%) was provided by Lipoid GmbH (Germany). Gallic acid and cholesterol (USP) were obtained from Sigma-Aldrich (USA). Folin-Ciocalteu phenol reagent, Triton X-100, anhydrous sodium carbonate, Mueller-Hinton broth (MHB), and Mueller-Hinton agar were supplied by Merck (Germany). All other chemicals were of analytical grade.

### 3.2. Determination of Honey Total Polyphenol Content

A Folin-Ciocalteu assay was performed to quantify total phenolic compounds in honey. Different solutions of gallic acid in distilled water (10 to 150 micrograms/mL) were prepared, and 200 microliters of each solution was mixed with 1 mL of 10% Folin-Ciocalteu reagent in water, followed by the addition of 800 microliters of a 7.5% (w/v) sodium carbonate solution and incubation for 30 minutes at room temperature in the dark. The absorbance (Abs) of the samples was measured at 760 nm using a UV-visible spectrophotometer (Cecil CE 2021, Cecil Instruments Ltd., UK) (16, 17). The total phenolic content (C) in honey was estimated from the gallic acid calibration curve (Abs = 0.0096C + 0.0315) and expressed as gallic acid equivalent (mg GA/g honey). As described later, for in vitro release studies, gallic acid was dissolved in phosphate-buffered saline instead of water to generate a calibration curve (Abs = 0.0104C + 0.00005).

### 3.3. Preparation of Liposomes

A thin-film hydration technique (18) was used to prepare five formulations of honey-loaded liposomes. Briefly, egg lecithin and cholesterol (20 micromol/mL total lipids at an 8:2 molar ratio) were dissolved in a chloroform:methanol mixture (2:1, v/v). The solvent was removed under vacuum at 50°C using a rotary evaporator (Heidolph, Germany). The lipid film was hydrated with honey solutions in water (initial or starting solutions) at concentrations of 5%, 15%, 30%, 50%, and 75% (w/v) at 50°C for 30 minutes, yielding formulations designated F1, F2, F3, F4, and F5, respectively. The liposomal suspensions were then resized using a probe sonicator (Topsonics, Iran) at 50% amplitude. A pulsed duty cycle (7 seconds on, 3 seconds off) was applied for a total treatment time of 12 minutes. Plain liposomes were prepared using the same procedure without honey (18).

### 3.4. Characterization of Plain and Loaded Liposomes

#### 3.4.1. Measurements of Particle Size and Zeta Potential

Liposome size distribution, polydispersity index, and zeta potential were assessed by dynamic light scattering (DLS) using a Zetasizer ZS instrument (Malvern Instruments, Malvern, UK) at room temperature.

#### 3.4.2. Determination of Entrapment Efficiency and Honey Content

The liposomal suspension was placed in a dialysis bag with a cutoff of 12 kDa and immersed in 200 mL of distilled water under stirring at 4°C at 100 rpm for 6 hours to separate free honey from the liposomes. After dialysis, 200 microliters of liposomes was destabilized by adding 20 microliters of 20% (w/v) Triton X-100 to disrupt the membranes. Total phenolic content was then analyzed using the Folin-Ciocalteu method (19, 20) and correlated with honey content (mg honey/mL formulation). Entrapment efficiency (EE%) was calculated using Equation 1:

Equation 1.

EE%=(entrappedhoney/initialhoney)×100

#### 3.4.3. Morphology

Field-emission scanning electron microscopy (FE-SEM, MIRA3 TESCAN, Czech Republic) was used to evaluate the surface characteristics of F5 as an example. A diluted liposomal formulation (1/10 in water) was placed on a glass slide, air-dried at room temperature, and analyzed.

### 3.5. In Vitro Honey Release from Liposomes

The in vitro release of honey from all formulations (F1 - F5) was evaluated using Franz diffusion cells with cellulose dialysis membranes with a cutoff of 12 kDa. The receptor compartment contained phosphate buffer at pH 7.4 at 37 ± 0.5°C. Samples were collected at specific time intervals (0.5, 1, 2, 4, 6, 8, 10, 24, 32, and 48 hours), and the receptor medium was replenished with fresh medium. Honey polyphenol content was determined using the Folin-Ciocalteu assay.

Release kinetics were analyzed by fitting the release data to mathematical models of zero-order (Equation 2), first-order (Equation 3), and Fickian diffusion for the nonswellable spherical model (Equation 4).

Equation 2.

$$M_{t}=K_{0}t$$

Equation 3.

$$\ln M_{\mathrm{rt}}=\ln M_{0}-K_{1}t$$

Equation 4.

$$M_{t}=K_{f}t^{0.43}$$

Where M_t_ represents the cumulative amount released at time t, t is time, and K is the rate constant corresponding to each model. M_0_ is the initial honey content, and M_rt_ is the amount of honey remaining in the system at time t, which is M_0_-M_t_.

### 3.6. Antibacterial Activity Studies

The minimum inhibitory concentration (MIC) of the formulations against *Staphylococcus aureus* ATCC 6538 and *Pseudomonas aeruginosa* ATCC 9027, obtained from the Iranian Research Organization for Science and Technology, Persian Type Culture Collection, Tehran, Iran, was determined using a microdilution method according to the Clinical and Laboratory Standards Institute guideline (21). Briefly, serial dilutions of honey and liposomal formulations were prepared in MHB using a 96-well microtiter plate. Bacterial suspensions were prepared from fresh colonies incubated at 37°C overnight, and their turbidity was adjusted to the 0.5 McFarland standard (1 x 10^8^ CFU/mL), which was then diluted in MHB to obtain an inoculum of approximately 5 × 10^6^ CFU/mL. Each well contained 100 microliters of samples at varying concentrations and 10 microliters of each bacterial suspension. Control wells containing samples in MHB (negative control) and bacteria in MHB (positive control) were included in each run, and the plates were incubated at 37°C for 24 hours. MIC values were defined as the lowest concentration that inhibited visible bacterial growth (22). In addition, the minimum bactericidal concentration (MBC) was determined by subculturing 10 microliters from each well without visible growth onto Mueller-Hinton agar medium and incubating at 37°C for 24 hours. The MBC was defined as the lowest concentration resulting in a negative subculture or no colony formation after the incubation period (23). All tests were performed in triplicate.

### 3.7. Statistical Analysis

All measurements were performed in triplicate. One-way analysis of variance and Tukey multiple comparison tests (P < 0.05) were used to compare the data using GraphPad Prism, Version 8.0.2 (GraphPad Software, Inc.).

## 4. Results

### 4.1. Determination of Total Polyphenol Content in Honey

A calibration curve was established using gallic acid as the reference standard for honey, enabling quantitative analysis of the phenolic compound content. In this study, the total polyphenol content was 0.363 ± 0.035 mg GA/g honey.

### 4.2. Characterization of Honey-Loaded Liposomes

#### 4.2.1. Size and Zeta Potential

Five different honey formulations were prepared, and particle size, polydispersity index, zeta potential, entrapment efficiency, and honey content were measured; the results are summarized in Table 1.

**Table 1.** Mean Particle Size, Polydispersity Index, Zeta Potential, Entrapment Efficiency (EE), and Honey Content (Final Honey Concentration) of Liposomal Formulations (Mean ± SD, N = 3) Prepared Using Different Starting Honey Solutions at an Initial Honey Concentration of 5% - 75% (w/v)

Samples	Honey% in Starting Solution	Z-average (nm)	Polydispersity Index	Zeta Potential (mV)	Entrapment Efficiency%	Final Honey Concentration (mg/mL)
**Plain liposome**	0	94.70 ± 2.20	0.34 ± 0.01	-16.30 ± 1.70	-	0
**F1**	5	129.83 ± 1.24	0.27 ± 0.01	-7.78 ± 0.35	21.79 ± 2.36	10.89 ± 1.18
**F2**	15	107.70 ± 2.86	0.28 ± 0.03	-7.32 ± 0.76	24.09 ± 3.43	36.14 ± 5.14
**F3**	30	104.57 ± 0.35	0.27 ± 0.01	-4.56 ± 1.60	33.49 ± 2.59	100.47 ± 7.78
**F4**	50	232.57 ± 15.86	0.15 ± 0.05	-3.59 ± 1.44	45.80 ± 1.94	229 ± 9.68
**F5**	75	282.53 ± 17.05	0.5 ± 0.05	-5.90 ± 0.42	37.73 ± 1.46	283 ± 11

The results showed that plain liposomes were smaller (94.7 nm) than the mean particle size of the honey-loaded formulations (Table 1). Notably, F5 and F4 (the two highest honey concentrations) were significantly larger than plain liposomes and F3, F2, and F1 (P < 0.0001).

For plain liposomes, the zeta potential was -16.30 mV. In the presence of honey, the surface charge of the liposomes was partially neutralized, decreasing to values less than -8 mV (Table 1).

#### 4.2.2. Entrapment Efficiency and Honey Content

Entrapment efficiency (EE) and honey content were determined for all formulations based on total phenolic content, which served as the bioactive component of honey. As shown in Table 1, increasing the honey concentration in the starting solution increased the final honey content of the formulations and, in most cases, the entrapment efficiency. Accordingly, F1 and F2 had the lowest EE values, approximately 22% and 24%, respectively (P > 0.05). F4 exhibited the highest entrapment efficiency, approximately 46% (P < 0.05). However, an inverse trend was observed when the initial honey concentration reached 75% (F5), resulting in an entrapment efficiency of approximately 38%. Therefore, the EE values of F5 and F3 were statistically similar (P > 0.05). These data were reproducible across different liposome batches assessed for EE.

#### 4.2.3. Morphology of Liposomes

The surface characteristics and morphology of F5 were examined by FE-SEM. The results showed spherical liposomes, along with some distorted spherical/angular particles of approximately 200 - 300 nm (Figure 1), which was in good agreement with the DLS data (Table 1), considering that DLS typically reports larger particle sizes (hydrodynamic diameter).

**Figure 1.**
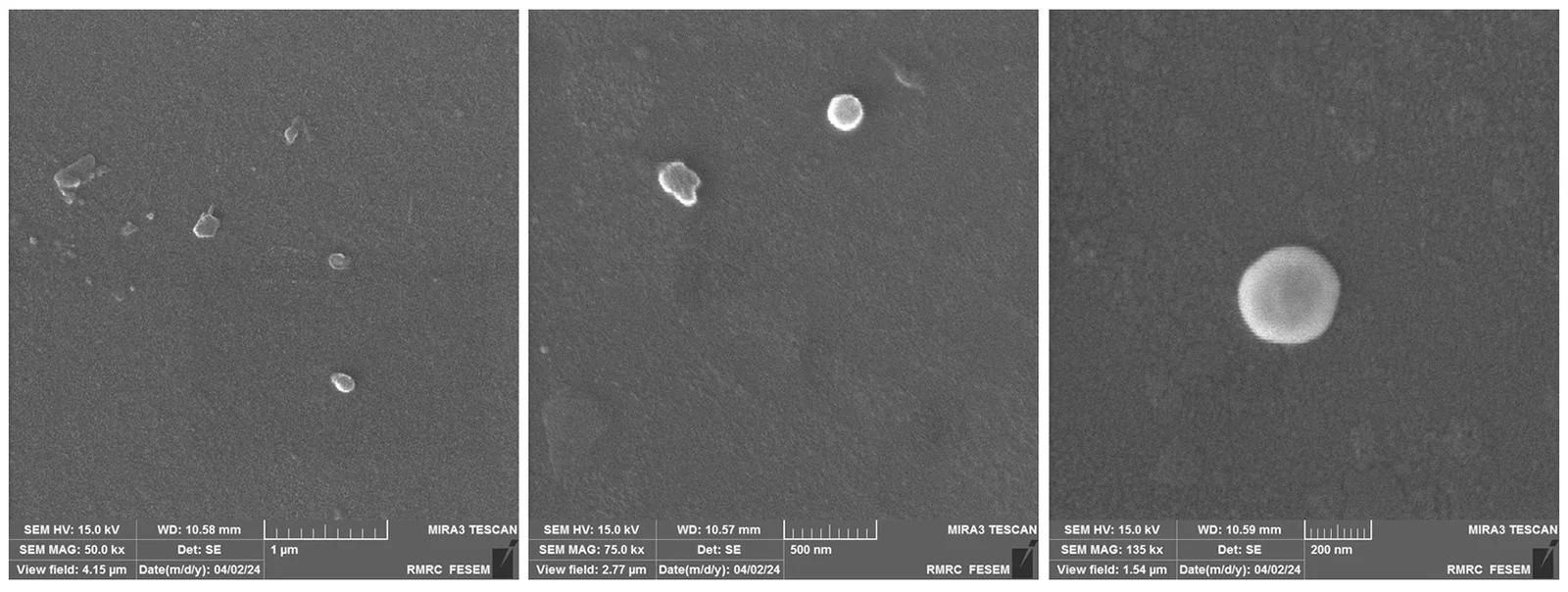
Sample surface morphologies of F5 as determined by FE-SEM. See text for more details.

### 4.3. In Vitro Release Studies

The in vitro release of honey from liposomes is shown in Figure 2. The release profile reached a near plateau after 6 - 10 hours (Figure 2). Over the first 10 hours, F1, F2, F3, F4, and F5 released approximately 15%, 20%, 24%, 50%, and 70% of the loaded honey, respectively. In contrast, honey was completely released from the 75% solution within 2 hours, which was significantly different from all liposomal formulations (P < 0.0001). At the 10-hour time point, no significant differences were observed among F1, F2, and F3, which were prepared with lower initial honey concentrations of 5%, 15%, and 30%, respectively. However, a significant (P < 0.05) increase in the percentage of honey released was observed for F4 compared with F1, F2, and F3. The highest release percentage (70%) was observed for F5, which had the highest starting honey concentration (75%, w/v), and was significantly higher than that of F4 (P < 0.05) and those of F1, F2, and F3 (P < 0.01).

**Figure 2.**
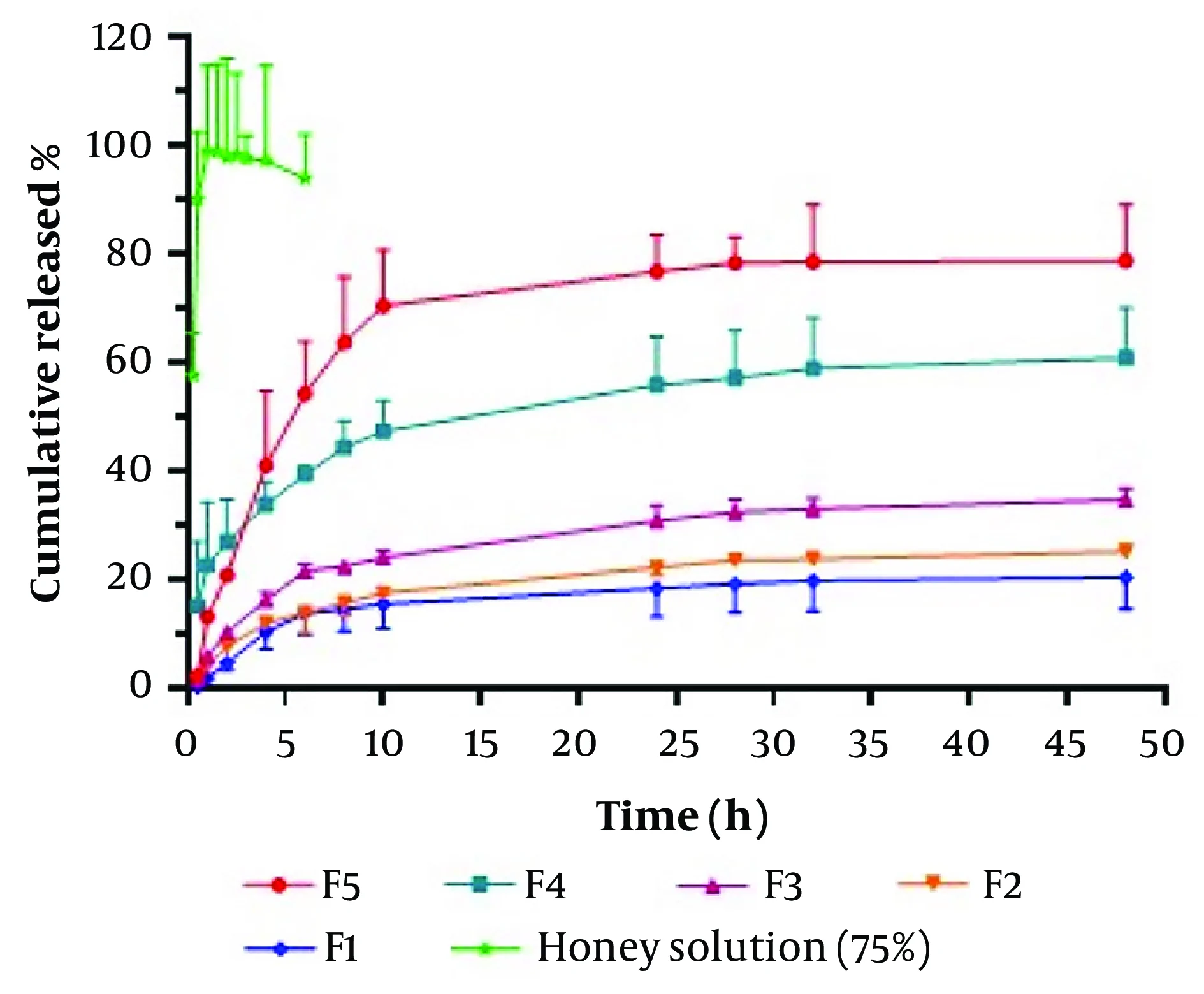
in vitro honey release profiles from liposomes containing different honey concentrations (F1 - F5) and 75% honey solution in phosphate-buffered saline (PBS) at pH 7.4 and 37°C over 48 hours (mean ± SD, n = 3).

Drug release data were also analyzed using three kinetic models (zero-order, first-order, and Fickian diffusion for spheres) over 0.5 - 10 hours. The coefficient of determination for each model is shown in Table 2. The most suitable kinetic model for honey-loaded liposomes (F1 - F4) was the Fickian model, indicating that honey release from liposomes (spheres) followed Fickian diffusion from spherical particles. For F5, the results were very close, and the system apparently favored first-order release kinetics. The results also indicated that the release constants/rates increased with increasing honey concentration across all models.

**Table 2.** Release Kinetic Constants (K) (Mean ± SD, N = 3) and Coefficient of Determination Values (R^2^) Obtained for Different Honey-Loaded Liposomes Using Various Kinetic Models

Formulation	Zero-order		First-order		Fickian diffusion for sphere	
	K_0_	R^2^	K_1_	R^2^	K_f_	R^2^
**F1**	1.66 ± 0.48	0.9053	-0.008 ± 0.002	0.9135	8.49 ± 2.42	0.9756
**F2**	1.59 ± 0.15	0.9126	-0.008 ± 0.001	0.9248	8.13 ± 0.80	0.9874
**F3**	2.29 ± 0.15	0.9016	-0.012 ± 0.001	0.9168	11.78 ± 0.61	0.9808
**F4**	3.15 ± 2.03	0.9419	-0.021 ± 0.013	0.9698	15.93 ± 10.03	0.9926
**F5**	7.08 ± 1.41	0.9586	-0.058 ± 0.019	0.9962	35.53 ± 7.49	0.9960

### 4.4. Antibacterial Activity Studies

Table 3 shows the MIC and MBC values for honey and liposomes determined by the broth microdilution method. The results are expressed based on the honey content in liposomes. The results indicated that, for honey, the most sensitive microorganism was *S. aureus*, with a minimum MIC of 62.5 mg/mL, whereas the MIC for the Gram-negative bacterium *P. aeruginosa* was 250 mg/mL.

**Table 3.** MIC and MBC of Honey, Honey-Loaded Liposomes, and Plain Liposomes (Based on Honey Content) Against *P. aeruginosa* and *S. aureus* Determined by the Microbroth Dilution Method ^a^

Samples	*Staphylococcus aureus*(mg/mL)		*Pseudomonas aeruginosa*(mg/mL)	
	MIC	MBC	MIC	MBC
**Honey**	62.5	62.5	250	250
**F1**	N/D	N/D	N/D	N/D
**F2**	N/D	N/D	N/D	N/D
**F3**	50.2	50.2	100.5	100.5
**F4**	57.2	57.2	57.2	57.2
**F5**	17.7	17.7	35.4	35.4
**Plain liposome**	N/D	N/D	N/D	N/D

^a^ The antibacterial activity is expressed as minimum inhibitory concentration (MIC) and minimum bactericidal concentration (MBC) (mg/mL) (n = 3). Abbreviation: N/D, Not detected.

In addition, as shown in Table 3, plain liposomes (without incorporated honey) and the liposomal systems F1 and F2 showed no antibacterial effect against Gram-positive or Gram-negative bacteria. However, F3 - F5 decreased both MIC and MBC relative to plain honey against both microorganisms in a concentration-dependent manner, with a greater effect against Gram-negative bacteria. The lowest MIC was 17.7 mg/mL, observed for F5 against *S. aureus*. The MBC values were very close to the MIC values for both bacterial strains, reflecting the bactericidal effect of the formulations.

## 5. Discussion

### 5.1. Characterization of Honey-Loaded Liposomes

Nanoparticle characteristics play a vital role in biopharmaceutical performance during drug delivery. Plain liposomes and F1, F2, and F3, prepared with 5%, 15%, and 30% initial honey concentrations, had sizes of approximately 90 - 130 nm, whereas sizes increased to 230 and 280 nm in F4 and F5, prepared with 50% and 75% initial honey concentrations, respectively. Aytekin et al. reported similar observations with propolis concentration, where increasing concentration led to a higher z-average of liposomes (14). In addition, a previous study found that increasing honey concentration resulted in larger nanofiber diameters because of the substantial honey content within the nanofibers (24).

Zeta potential is a critical parameter related to the stability of colloidal systems. In the present study, adding honey to the liposomes increased the zeta potential from -16 mV in plain liposomes to values between -4 and -8 mV in honey-containing liposomes. This behavior could be attributed to partial neutralization of anionic phospholipid head groups by loaded honey constituents, such as phenolic acids and flavonoids, and may indicate that certain honey compounds, such as polyphenols, reside within the liposomal membrane (25). Previous studies have also indicated that interactions between certain phenolic compounds and phospholipids can reduce the surface charge of liposomes (19, 26).

### 5.2. Entrapment Efficiency

Based on the results, the honey content and entrapment efficiency of F1 - F5 were influenced by the initial honey concentration. For F5, entrapment efficiency decreased compared with F4. This decrease might be due to the higher viscosity of the medium and lower water content at a higher honey concentration (75% honey), which might reduce molecular motion and lead to decreased entrapment (27, 28). In addition, the results showed larger particle sizes in F5, indicating a lower number of particles. Moreover, excessive honey insertion might have saturated the lipid bilayers. A previous study by Ramli et al. reported that when propolis extract concentration was added in excess, instability of the liposome suspension was observed (29).

### 5.3. Release Study

The results showed that entrapment of honey in liposomes decreased the release rate compared with honey solution (Figure 2), indicating delayed/extended release behavior. In liposomal systems, increased honey concentration increased the release rate and amount, although the differences were not significant for F1 - F3 (P > 0.05). This finding may suggest that liposomes act as a honey reservoir and that, as the honey concentration reaches higher values in F4 and F5, the reservoir (binding sites) becomes filled/saturated, leaving more free honey available for release from the liposomes. In addition, F1, F2, and F3 reached the plateau faster than F4 and F5, possibly because of their lower honey concentrations.

### 5.4. Antibacterial Study

The results showed that F3-F5 had lower MIC and MBC values than pure honey, up to 3.5-fold against *S. aureus* and 7-fold against *P. aeruginosa*, based on honey content. These improvements may be due to bacterial membrane perturbation or higher uptake of liposomal systems (14). According to previous studies, honey contains phenolic and flavonoid compounds that could contribute to its antibacterial activity. However, because of their high molecular weight and hydrophilicity, these materials could have difficulties in penetrating the bacterial outer membrane sufficiently, especially in Gram-negative bacteria (5, 30). Gram-positive bacteria lack this outer membrane layer; therefore, as confirmed by our results, honey is effective against *S. aureus*, whereas Gram-negative bacteria are less sensitive to honey, possibly because their outer membrane resists the entry of these active compounds into the cell. It seems reasonable that, by increasing permeation through this barrier, which may be achieved using liposomes, this limitation was reduced, and honey became 7-fold more effective against this type of bacteria, in agreement with previous findings for some antibiotics (22, 30).

In support of our study, incorporation of propolis into liposomes led to greater antibacterial activity against microorganisms than propolis solution by affecting the bacterial cell wall and increasing membrane permeability (14). Such activity may have contributed to the effects of liposomes on bacteria observed here.

The literature consistently highlights the beneficial antibacterial properties of honey, primarily attributed to its polyphenols (31-33). Investigation of a novel drug delivery system using selenium-based nanoparticles for honey polyphenols (HP) demonstrated that it not only served as an efficient delivery system but also significantly enhanced HP bioavailability. Consequently, this enhancement contributed to the antibiofilm activity of HP against *Pseudomonas aeruginosa* (5). Therefore, honey polyphenols play a crucial role in its antimicrobial effects, and this effect can be improved by appropriate drug delivery systems, as shown in the present investigation.

### 5.5. Conclusions

Liposomes are suitable nanoformulations for the delivery of multicomponent herbal systems. In this study, honey-loaded liposomes with different initial honey concentrations were formulated and characterized for size, zeta potential, entrapment efficiency, and antibacterial effects. The results showed that honey concentration had a marked effect on liposome characteristics, including size, zeta potential, entrapment efficiency, and release behavior. In vitro antibacterial studies showed that honey-loaded liposomes were up to 3.5-fold and 7-fold more effective than honey solution against Gram-positive *S. aureus* and Gram-negative *P. aeruginosa*, respectively.

Therefore, encapsulation of honey in liposomes could be a promising technique for delivering bioactive ingredients such as honey to the target site in biological systems. Future research should include detailed in vivo studies in animal models to further establish the efficacy of the developed liposomes as a potential honey delivery system.

## Data Availability

The dataset presented in the study is available on request from the corresponding author during submission or after publication.
